# Reinforcement Learning Explains Conditional Cooperation and Its Moody Cousin

**DOI:** 10.1371/journal.pcbi.1005034

**Published:** 2016-07-20

**Authors:** Takahiro Ezaki, Yutaka Horita, Masanori Takezawa, Naoki Masuda

**Affiliations:** 1 Research Center for Advanced Science and Technology, The University of Tokyo, Meguro-ku, Tokyo, Japan; 2 Japan Society for the Promotion of Science, Kojimachi, Chiyoda-ku, Tokyo, Japan; 3 National Institute of Informatics, Hitotsubashi, Chiyoda-ku, Tokyo, Japan; 4 JST, ERATO, Kawarabayashi Large Graph Project, c/o Global Research Center for Big Data Mathematics, NII, Chiyoda-ku, Tokyo, Japan; 5 Department of Behavioral Science, Hokkaido University, Kita-ku, Sapporo, Japan; 6 Center for Experimental Research in Social Sciences, Hokkaido University, Kita-ku, Sapporo, Japan; 7 Department of Engineering Mathematics, University of Bristol, Clifton, Bristol, United Kingdom; University of California, Irvine, UNITED STATES

## Abstract

Direct reciprocity, or repeated interaction, is a main mechanism to sustain cooperation under social dilemmas involving two individuals. For larger groups and networks, which are probably more relevant to understanding and engineering our society, experiments employing repeated multiplayer social dilemma games have suggested that humans often show conditional cooperation behavior and its moody variant. Mechanisms underlying these behaviors largely remain unclear. Here we provide a proximate account for this behavior by showing that individuals adopting a type of reinforcement learning, called aspiration learning, phenomenologically behave as conditional cooperator. By definition, individuals are satisfied if and only if the obtained payoff is larger than a fixed aspiration level. They reinforce actions that have resulted in satisfactory outcomes and anti-reinforce those yielding unsatisfactory outcomes. The results obtained in the present study are general in that they explain extant experimental results obtained for both so-called moody and non-moody conditional cooperation, prisoner’s dilemma and public goods games, and well-mixed groups and networks. Different from the previous theory, individuals are assumed to have no access to information about what other individuals are doing such that they cannot explicitly use conditional cooperation rules. In this sense, myopic aspiration learning in which the unconditional propensity of cooperation is modulated in every discrete time step explains conditional behavior of humans. Aspiration learners showing (moody) conditional cooperation obeyed a noisy GRIM-like strategy. This is different from the Pavlov, a reinforcement learning strategy promoting mutual cooperation in two-player situations.

## Author Summary

Laboratory experiments using human participants have shown that, in groups or contact networks, humans often behave as conditional cooperator or its moody variant. Although conditional cooperation in dyadic interaction is well understood, mechanisms underlying these behaviors in group or networks beyond a pair of individuals largely remain unclear. In this study, we show that players adopting a type of reinforcement learning exhibit these conditional cooperation behaviors. The results are general in the sense that the model explains experimental results to date obtained in various situations. It explains moody conditional cooperation, which is a recently discovered behavioral trait of humans, in addition to traditional conditional cooperation. It also explains experimental results obtained with both the prisoner’s dilemma and public goods games and with different population structure. Crucially, our model assumes that individuals do not have access to information about what other individuals are doing such that they cannot explicitly condition their behavior on how many others have previously cooperated. Thus, our results provide a proximate and unified understanding of these experimentally observed patterns.

## Introduction

Humans very often cooperate with each other when free-riding on others’ efforts is ostensibly lucrative. Among various mechanisms enabling cooperation in social dilemma situations, direct reciprocity, i.e., repeated interaction between a pair of individuals, is widespread. If individuals will repeatedly interact, they are motivated to keep on cooperation because no cooperation would invite retaliation by the peer in the succeeding interactions [1, 2]. Past theoretical research using the two-player prisoner’s dilemma game (PDG) identified tit-for-tat (TFT) [2], generous TFT [3], a win-stay lose-shift strategy often called Pavlov [4–6] as representative strong competitors in the repeated two-player PDG.

Direct reciprocity in larger groups corresponds to the individual’s action rule collectively called the conditional cooperation (CC), a multiplayer variant of TFT. By definition, an individual employing CC would cooperate if a large amount of cooperation has been made by other group members. In the present study, we study a reinforcement learning model. Depending on the parameter values, the outcome of the learning process shows CC patterns and their variant that have been observed in behavioral experiments.

In fact, the following evidence suggests that the concept and relevance of CC are much more nuanced than in the case of dyadic interactions, calling for examinations. First, early theoretical studies have concluded that CC in the multiplayer PDG is unstable as the group size increases [7, 8]. In addition, CC assumed in these and follow-up studies is a threshold behavior (i.e., players cooperate when the number of peers that have cooperated the last time exceeds a prescribed threshold). However, CC patterns and their variants observed in the extant experiments are gradual rather than a threshold behavior [9–17].

Second, the public goods game (PGG) models social dilemmas occurring in a group beyond a pair of individuals. In the repeated PGG, CC has been observed in laboratory experiments with human participants [9–12, 17] and in real society [18]. By definition, an individual adopting CC increases the amount of cooperation when others have made large contributions the last time. CC in the repeated PGG with two or more players is theoretically stable under some conditions [19–24]. However, these conditions are not generous and how they connect to the experimental results is not clear. This situation contrasts to that of the two-player, discrete-action PDG mentioned previously, where conditions under which direct reciprocity occurs and strategies enabling them are well characterized [2, 3, 5].

Third, recent experiments have discovered that humans show moody conditional cooperation (MCC) behavior in the repeated PDG game played on contact networks [13–16]. MCC is defined as follows. MCC is the same as CC if the player has cooperated the last time. If the player has defected the last time, a player adopting MCC decides on the action without taking into account what the neighbors in the contact network have done previously. In this sense, the player’s action rule is moody. The genesis of MCC is not well understood. First, evolutionary dynamics do not promote MCC behavior [15, 25]. Second, non-evolutionary numerical simulations assuming MCC do not intend to explain why MCC emerges or is stable [14, 15, 26]. Third, a numerical study employing reinforcement learning [25] has MCC behavior built in into the model in the sense that MCC occurs whenever cooperation is sustained (see Discussion for more).

In this article, we provide an account for experimentally observed CC and MCC patterns using a family of reinforcement learning called the aspiration learning [27–36]. In reinforcement learning, players satisfice themselves rather than maximize the payoff in the sense that a player increases and decreases the likelihood of the behavior that has yielded a large and small reward, respectively. In aspiration learning, players are satisfied if and only if the obtained payoff is larger than a threshold. Because the probability to select the behavior, such as cooperation, is dynamically updated in every discrete time step, aspiration learning is different from a conditional strategy in general.

Our main conclusion that reinforcement learning explains CC and MCC resembles that of a previous study [25]. However, the present study is radically different from Ref. [25] in the following aspects. First, as stated above, MCC behavior is an assumed mode of the model proposed in Ref. [25]. In the present model, players myopically adjust the unconditional probability of cooperation depending on the previous action and reward, as in previous aspiration learning models [28, 29, 31, 32, 37–39]. Second, the present model is also simpler, even without assuming players to be aware of the amount of cooperation carried out nearby or to explicitly implement conditional strategies.

### Model

We place a player obeying the reinforcement learning rule on each node of the square lattice with 10 × 10 nodes with periodic boundary conditions. However, the following results do not require particular network structure (Fig A in S1 Text). Each player is involved in the two-player PDG against each of the four neighbors on the network. The game is also interpreted as a PGG played in the group composed of the player and all neighbors submitting binary decisions [40]. The game is repeated over *t*_max_ rounds. We set *t*_max_ = 25 unless otherwise stated.

**Fig 1:**
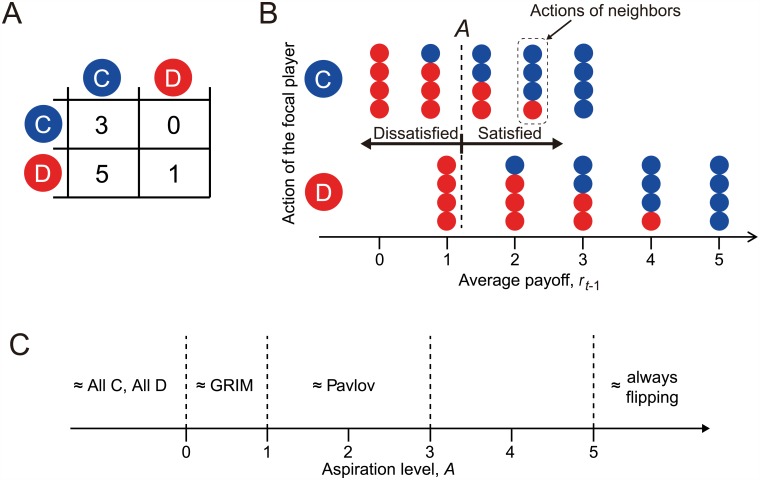
Behavior of the aspiration learner in the repeated PD game. (A) Payoff matrix. The payoff values for the row player are shown. (B) Concept of satisficing in the aspiration-based reinforcement learning model. The payoff values shown on the horizontal axis are those for the focal player. (C) Relationship between the aspiration level, *A*, and the approximate (un)conditional strategy, given the payoff matrix shown in (A).

Each player selects either to cooperate (C) or defect (D) in each round (Fig 1A). The submitted action (i.e., C or D) is used consistently against all the neighbors. In other words, a player is not allowed to cooperate with one neighbor and defect against another neighbor in the same round. If both players in a pair cooperate, both players gain payoff *R* = 3. If both defect, both gain *P* = 1. If a player cooperates and the other player defects, the defector exploits the cooperator such that the cooperator and defector gain *S* = 0 and *T* = 5, respectively.

Each player is assumed to update the intended probability to cooperate, *p*_*t*_, according to the Bush-Mosteller (BM) model of reinforcement learning [27–29, 32, 39] as follows:

$$p_{t}=\left\{\begin{array}{cc} p_{t-1}+\left(1-p_{t-1}\right)s_{t-1} & (a_{t-1}=\text{C},s_{t-1}\geq 0),\\ p_{t-1}+p_{t-1}s_{t-1} & (a_{t-1}=\text{C},s_{t-1}<0),\\ p_{t-1}-p_{t-1}s_{t-1} & (a_{t-1}=\text{D},s_{t-1}\geq 0),\\ p_{t-1}-\left(1-p_{t-1}\right)s_{t-1} & (a_{t-1}=\text{D},s_{t-1}<0), \end{array}\right.$$ (1)

where *a*_*t*−1_ is the action in the (*t*− 1)th round, and *s*_*t*−1_ is the stimulus that drives learning (−1 < *s*_*t*−1_ < 1). The current action is reinforced and suppressed if *s*_*t*−1_ > 0 and *s*_*t*−1_ < 0, respectively. For example, the first line on the right-hand side of Eq (1) states that the player increases the probability to cooperate if it has cooperated and been satisfied in the previous round. The multiplicative factor (1 − *p*_*t*−1_) is imposed to respect the constraint *p*_*t*_ < 1.

The stimulus is defined by

$$s_{t-1}=\tanh [\beta (r_{t-1}-A)],$$ (2)

where *r*_*t*−1_ is the payoff to the player in round *t* − 1, averaged over the four neighboring players, *A* is the aspiration level, and *β*(> 0) controls the sensitivity of *s*_*t*−1_ to *r*_*t*−1_ − *A* [39]. The player is satisfied and dissatisfied if *r*_*t*−1_ − *A* > 0 (i.e., *s*_*t*−1_ > 0) and *r*_*t*−1_ − *A* < 0 (i.e., *s*_*t*−1_ < 0), respectively (Fig 1B). The so-called Pavlov strategy corresponds to *β* = ∞ and *P* < *A* < *R* [4, 5] (Fig 1C). The so-called GRIM strategy, which starts with cooperation and turns into permanent defection (if without noise) once the player is defected [2, 41], corresponds to *β* = ∞ and *S* < *A* < *R* [38]. When *β* < ∞, which we assume, the behavior realized by the BM model is not an exact conditional strategy such as Pavlov or GRIM, but an approximate one. Unlike some previous studies in which *A* adaptively changes over time [32, 37–39], we assume that *A* is fixed.

In each round, each player is assumed to misimplement the decision with probability *ϵ* [5, 6, 39]. Therefore, the actual probability to cooperate in round *t* is given by ${\tilde{p}}_{t}\equiv p_{t}\left(1-\epsilon \right)+\left(1-p_{t}\right)\epsilon$. We set *ϵ* = 0.2 and the initial probability of cooperation *p*_1_ = 0.5 unless otherwise stated.

## Results

### Prisoner’s dilemma game

**Fig 2:**
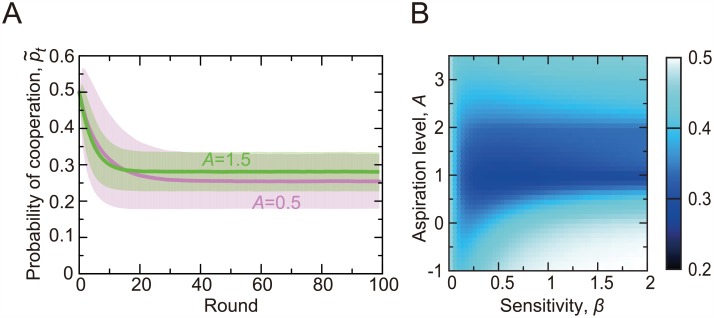
Probability of cooperation in the repeated PDG game on the square lattice having 10 × 10 nodes. (A) Mean time courses of the actual probability of cooperation, ${\tilde{p}}_{t}$. The lines represent the actual probability of cooperation averaged over the 10^2^ players and 10^3^ simulations. We set *β* = 0.2 and *A* = 0.5. The shaded regions represent the error bar calculated as one standard deviation. (B) Probability of cooperation for various values of the sensitivity of the stimulus to the reward, *β*, and the aspiration level, *A*. The shown values are averages over the 10^2^ players, the first *t*_max_ = 25 rounds, and 10^3^ simulations.

For *A* = 0.5 and *A* = 1.5, the realized probability of cooperation, ${\tilde{p}}_{t}$, averaged over the players and simulations is shown in Fig 2A up to 100 rounds. Due to a relatively large initial probability of cooperation, *p*_1_ = 0.5, ${\tilde{p}}_{t}$ drops within the first ≈20 rounds and stays at the same level afterwards for both *A* values. This pattern is roughly consistent with behavioral results obtained in laboratory experiments [13–16, 42].

For a range of the two main parameters, the sensitivity of the stimulus to the reward (i.e., *β*) and the aspiration level setting the satisfaction threshold for players (i.e., *A*), ${\tilde{p}}_{t}$ averaged over the first 25 rounds is shown in Fig 2B. The figure indicates that cooperation is frequent when *β* is large, which is consistent with the previous results [39], and when *A* is less than ≈1. The probability of cooperation is also relatively large when *A* is larger than ≈2. In this situation, defection leads to an unsatisfactory outcome unless at least two out of the four neighbors cooperate (Fig 1B). Because this does not happen often, a player would frequently switch between defection and cooperation, leading to ${\tilde{p}}_{t}\approx 0.4$.

The results shown in Fig 2B were largely unchanged when we varied *t*_max_ and *ϵ* (Fig B in S1 Text).

**Fig 3:**
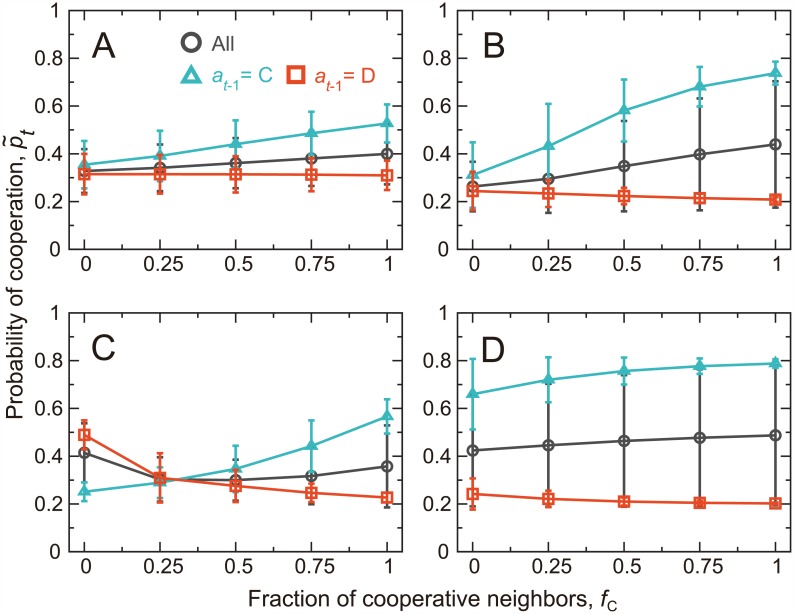
CC and MCC in the repeated PDG on the square lattice. The actual probability of cooperation, ${\tilde{p}}_{t}$, is plotted against the fraction of cooperative neighbors in the previous round, *f*_C_. The error bars represent the mean ± standard deviation calculated on the basis of all players, *t*_max_ = 25 rounds, and 10^3^ simulations. The circles represent the results not conditioned on *a*_*t*−1_. The triangles and the squares represent the results conditioned on *a*_*t*−1_ = C and *a*_*t*−1_ = D, respectively. We set (A) *β* = 0.1 and *A* = 0.5, (B) *β* = 0.4 and *A* = 0.5, (C) *β* = 0.4 and *A* = 2.0, and (D) *β* = 0.4 and *A* = −1.0.

The probability of cooperation, ${\tilde{p}}_{t}$, is plotted against the fraction of cooperating neighbors in the previous round, denoted by *f*_C_, for *A* = 0.5 and two values of *β* in Fig 3A and 3B. The results not conditioned on the action of the player in the previous round are shown by the circles. The player is more likely to cooperate when more neighbors cooperate, consistent with CC patterns reported in experiments with the PDG on the square lattice [42]. CC is particularly pronounced at a large value of *β* (Fig 3B as compared to Fig 3A).

The relationship between ${\tilde{p}}_{t}$ and *f*_C_ conditioned on the last action of the focal player, denoted by *a*_*t*−1_, is shown by the triangles and squares. We observe clear MCC patterns, particularly for a large *β*. In other words, players that have previously cooperated (i.e., *a*_*t*−1_ = C) show CC, whereas the probability of cooperation stays constant or mildly decreases as *f*_C_ increases when the player has previously defected (i.e., *a*_*t*−1_ = D). These MCC patterns are consistent with the extant experimental results [13–16].

In the experiments, MCC has also been observed for different population structure such as the scale-free network [16] and a dynamically changing network [14]. We carried out numerical simulations on the regular random graph (i.e., random graph in which all nodes have the same degree, or the number of neighbors) with degree four and the well-mixed group of five players in which each player had four partners. The results remained qualitatively the same as those for the square lattice, suggesting robustness of the present numerical results with respect to the network structure (Fig A in S1 Text). Spatial or network reciprocity is not needed for the present model to show MCC patterns.

A different aspiration level, *A*, produces different patterns. CC and MCC patterns are lost when we set *A* = 2 (Fig 3C), with which the dependence of ${\tilde{p}}_{t}$ on *f*_C_ is small, and ${\tilde{p}}_{t}$ when no neighbor has cooperated in the previous round (i.e., *f*_C_ = 0) is larger for *a*_*t*−1_ = D (squares in Fig 3C) than for *a*_*t*−1_ = C (triangles). The latter pattern in particular contradicts the previous behavioral results [13–16]. CC and MCC patterns are mostly lost for *A* = −1 as well (Fig 3D). With *A* = −1, the BM player is satisfied by any outcome such that any action is reinforced except for the action implementation error. Therefore, the behavior is insensitive to the reward, or to *f*_C_.

**Fig 4:**
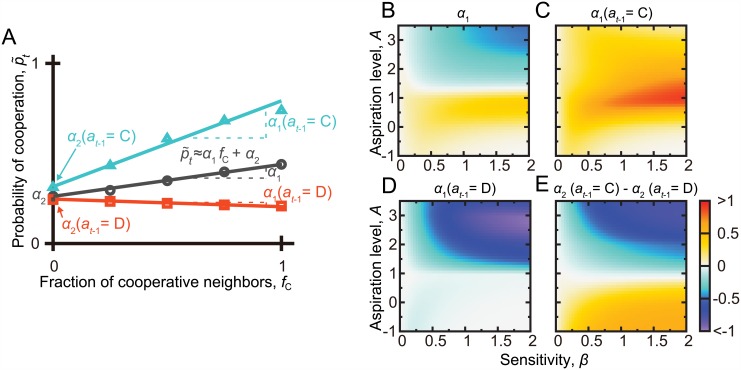
Search of CC and MCC patterns in the repeated PDG on the square lattice. (A) Schematic of the linear fit, ${\tilde{p}}_{t}\approx \alpha_{1}f_{C}+\alpha_{2}$. (B) Slope *α*_1_ of the linear fit when not conditioned on the focal player’s previous action, *a*_*t*−1_. (C) *α*_1_ when conditioned on *a*_*t*−1_ = C. (D) *α*_1_ when conditioned on *a*_*t*−1_ = D. (E) Difference between the intercept, *α*_2_, obtained from the linear fit conditioned on *a*_*t*−1_ = C and that conditioned on *a*_*t*−1_ = D. For each combination of the *β* and *A* values, a linear fit was obtained by the least-squares method on the basis of the 10^2^ players, *t*_max_ = 25 rounds, and 10^3^ simulations, yielding 2.5 × 10^6^ samples in total.

To assess the robustness of the results, we scanned a region in the *β* − *A* parameter space. For each combination of *β* and *A* values, we performed linear least-square fits to the relationship between the mean ${\tilde{p}}_{t}$ and *f*_C_, estimating ${\tilde{p}}_{t}\approx \alpha_{1}f_{C}+\alpha_{2}$ (Fig 4A). CC is supported if the obtained slope *α*_1_ is positive when unconditioned on *a*_*t*−1_ (circles in Fig 3). MCC is supported if *α*_1_ is positive when *a*_*t*−1_ = C (triangles in Fig 3) and negative or close to zero when *a*_*t*−1_ = D (squares in Fig 3). Intercept *α*_2_ is equal to the value of ${\tilde{p}}_{t}$ when no neighbor has cooperated in the previous round. The behavioral results suggest that *α*_2_ is larger when conditioned on *a*_*t*−1_ = C than on *a*_*t*−1_ = D [13–16].

Fig 4B indicates that the slope *α*_1_ unconditioned on *a*_*t*−1_ is positive, producing CC, when *A* ≤ 1 and *β* is larger than ≈0.25. However, *α*_1_ is less positive when *A* is extremely small, i.e., smaller than ≈0. When conditioned on *a*_*t*−1_ = C, *α*_1_ is positive, consistent with the MCC patterns, except when *β* is larger than ≈0.5 and *A* is smaller than ≈0 (Fig 4C). When conditioned on *a*_*t*−1_ = D, *α*_1_ is close to zero when *A* ≤ 1 and substantially negative when *A* ≥ 1 (Fig 4D). The difference in the value of *α*_2_, the intercept of the linear fit, between the cases *a*_*t*−1_ = C and *a*_*t*−1_ = D is shown in Fig 4E. The figure indicates that this value is non-negative, consistent with the experimental results, only when *A* < 1. To conclude, CC and MCC patterns consistent with the behavioral results are produced when 0 < *A* < 1 and *β* is not too small. We also confirmed that a different implementation of the BM model [32] produced CC and MCC patterns when *A* < 1 (Fig C in S1 Text).

The BM model with *P* < *A* < *R*, i.e., 1 < *A* < 3, corresponds to the Pavlov strategy, which is a strong competitor and facilitator of cooperation in the repeated PDG [4, 5]. Our results do not indicate that the Pavlov strategy explains CC and MCC patterns. In fact, the BM model with *S* < *A* < *P* (i.e., 0 < *A* < 1), which is a noisy GRIM-like reinforcement learning, robustly produces CC and MCC patterns. It should be noted that, a noisy GRIM strategy without reinforcement learning components does not produce CC and MCC patterns (Fig D in S1 Text). This result suggests an active role of reinforcement learning rather than merely conditional strategies such as the noisy GRIM.

### Public goods game

CC behavior has been commonly observed for humans engaged in the repeated PGG in which participants make a graded amount of contribution [9–11, 43–45]. It should be noted that the player’s action is binary in the PDG. In accordance with the setting of previous experiments [10], we consider the following repeated PGG in this section. We assume that four players form a group and repeatedly play the game. In each round, each player receives one monetary unit and determines the amount of contribution to a common pool, denoted by *a*_*t*_ ∈ [0, 1]. The sum of the contribution over the four players is multiplied by 1.6 and equally redistributed to them. Therefore, the payoff to a player is equal to $1-a_{t}+0.4(a_{t}+\sum_{j=1}^{3}{\tilde{a}}_{j,t})$, where ${\tilde{a}}_{j,t}$ is the contribution by the *j*th other group member in round *t*. The Nash equilibrium is given by no contribution by anybody, i.e., $a_{t}={\tilde{a}}_{j,t}=0$ (1 ≤ *j* ≤ 3).

We simulated the repeated PGG in which players implemented a variant of the BM model (see Materials and Methods). Crucially, we introduced a threshold contribution value *X* above which the action was regarded to be cooperative. In other words, an amount of contribution *a*_*t*_ ≥ *X* and *a*_*t*_ < *X* are defined to be cooperation and defection, respectively. Binarization of the action is necessary for determining the behavior to be reinforced and that to be anti-reinforced.

**Fig 5:**
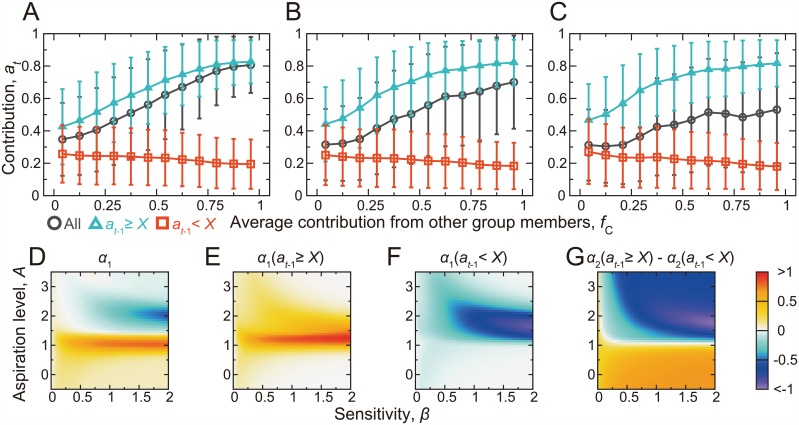
CC and MCC patterns in the repeated PGG in a group of four players. (A)–(C) Contribution by a player (i.e., *a*_*t*_) conditioned on the average contribution by the other group members in the previous round (i.e., *f*_C_). We set *β* = 0.4 and *A* = 0.9. (A) *X* = 0.3, (B) *X* = 0.4, and (B) *X* = 0.5. The circles represent the results not conditioned on *a*_*t*−1_. The triangles and the squares represent the results conditioned on *a*_*t*−1_ ≥ *X* and *a*_*t*−1_ < *X*, respectively. (D) Slope *α*_1_ of the linear fit, *a*_*t*_ ≈ *α*_1_ *f*_C_ + *α*_2_, when not conditioned on *a*_*t*−1_. (E) *α*_1_ when conditioned on *a*_*t*−1_ ≥ *X*. (F) *α*_1_ when conditioned on *a*_*t*−1_ < *X*. (G) Difference between *α*_2_ obtained from the linear fit conditioned on *a*_*t*−1_ ≥ *X* and that conditioned on *a*_*t*−1_ < *X*. The mean and standard deviation in (A)–(C) and the linear fit used in (D)–(G) were calculated on the basis of the four players, *t*_max_ = 25 rounds, and 2.5 × 10^4^ simulations, yielding 2.5 × 10^6^ samples in total.

In Fig 5, the contribution by a player, *a*_*t*_, averaged over the players, rounds, and simulations is plotted against the average contribution by the other group members, which is again denoted by *f*_C_(0 ≤ *f*_C_ ≤ 1). We observe CC behavior for this parameter set when *X* = 0.3 and 0.4 (circles in Fig 5A and 5B, respectively). CC patterns are weak for *X* = 0.5 (Fig 5C). The average contribution by a player as a function of *f*_C_ and the action of the focal player in the previous round is shown by the triangles and squares in Fig 5A–5C. We find MCC patterns. CC and MCC shown in Fig 5 are robustly observed if *β* is larger than ≈0.2, *A* ≤ 1, and 0.1 ≤ *X* ≤ 0.4 (Fig 5D–5G and Fig E in S1 Text).

Directional learning is a reinforcement learning rule often applied to behavioral data in the PGG [46, 47] and the PDG [48]. By definition, a directional learner keeps increasing (decreasing) the contribution if an increase (decrease) in the contribution in the previous round has yielded a large reward. In a broad parameter region, we did not find CC or MCC behavior with players obeying the directional learning rule (Fig F in S1 Text). The present BM model is simpler and more accurate in explaining the experimental results in terms of CC and MCC patterns than directional learning is.

### Presence of free riders

So far, we have assumed that all players are aspiration learners. Empirically, strategies depend on individuals in the repeated PDG [13, 15, 16] and PGG [9, 10, 18]. In particular, a substantial portion of participants in the repeated PGG, varying between 2.5% and 33% depending on experiments, is free rider, i.e., unconditional defector [9, 43, 49, 50]. Therefore, we performed simulations when BM players and unconditional defectors were mixed. We found that the CC and MCC patterns measured for the learning players did not considerably alter in both PDG and PGG when up to half the players were assumed to be unconditional defectors (Fig G in S1 Text).

## Discussion

We have provided compelling numerical evidence that the BM model, a relatively simple aspiration-based reinforcement learning model that has been employed in various decision making tasks [27–29, 31–39], explains CC and MCC patterns. On one hand, aspiration learning has offered a proximate mechanism for cooperation [28, 29, 31, 32, 37–39]. On the other hand, conditional cooperation in the repeated PGG [9–11, 43–45] and its moody variant in the repeated PDG on networks [13–16] have been consistently observed. Here we provided a connection between aspiration learning and conditional cooperation. Our choice of the parameter values including the number of rounds, the size of the group or neighborhood, and the payoff values, supports the comparison of the present numerical data with the results of behavioral experiments.

We are not the first to provide this link. Cimini and Sánchez have shown that MCC emerges from a BM model [25]. The current results significantly depart from theirs and are fundamentally new as follows.

First, MCC is built in into their model in the sense that every outcome except for a population of unconditional defectors implies MCC patterns. In their model, the linear relationship *p*_*t*_ = *α*_1_ *f*_C_ + *α*_2_ after the focal player’s cooperation, where *p*_*t*_ is the probability of cooperation and *f*_C_ is the fraction of cooperation in the neighborhood in the previous round, adaptively changes according to the BM model dynamics. In fact, *α*_1_ and *α*_2_ are simultaneously updated under a constraint and take a common value after a transient (S1 Text), consistent with their numerical results (Fig 2 in [25]). This relationship yields *p*_*t*_ = *α*_1_(*f*_C_ + 1), implying MCC whenever *α*_1_ > 0. When *α*_1_ = 0, we obtain *p*_*t*_ = 0, i.e., unconditional defection. In contrast, players in our model directly adapt the unconditional probability of cooperation without knowing *f*_C_ such that there is no room for players to explicitly learn the MCC rule. Therefore, our approach is inherently bottom-up.

Second, our model is cognitively less taxing than the Cimini-Sánchez model. In their model, a player refers to *f*_C_ and updates the action rule based on its own actions in the last two rounds. Depending on the action that the player has submitted in the second last round, the parameters in one of the two subrules ((*p*, *r*) or *q* in [25]) are updated. In contrast, as already mentioned, players do not refer to *f*_C_ in our model. They only refer to their own reward and action in the previous round. A player simply increases or decreases the unconditional probability of cooperation in the next round depending on the amount of satisfaction, as assumed in the previous experimental [28] and theoretical [29, 32, 37–39] studies applying aspiration-based reinforcement learning models to social dilemma games.

In Ref. [25], the Pavlov rather than GRIM rule produced MCC patterns. Our results were the opposite. With Pavlov, CC behavior is lost in our simulations (Figs 4B and 5D). In addition, a Pavlov player cooperates more often after it has defected than cooperated in the last round (Figs 4E and 5G), qualitatively contradicting the experimental results. This inconsistency with Pavlov persists even if we use the Macy-Flache reinforcement learning model as in [25] (Fig C in S1 Text). MCC is intuitively associated with GRIM, not Pavlov, for the following reason. Consider the two-person PDG for simplicity and a player obeying MCC. The player and has obtained payoff *R* (by mutual cooperation; *f*_C_ = 1), the player would cooperate in the next round. If the same MCC player has obtained payoff *S* (by the player’s unilateral cooperation; *f*_C_ = 0), the player would defect in the next round. If the player has obtained payoff *P* or *T* (by the player’s defection, i.e., *a*_*t*−1_ = D), the player would next submit *a*_*t*_ (= C or D) independently of the previously obtained payoff (i.e., *P* or *T*). If *a*_*t*_ = C, the player has flipped the action because *a*_*t*−1_ = D. This MCC behavior is not realizable by the aspiration learning because it requires *S*, *P*, *T* < *A* < *R*, which contradicts the payoff of the PDG, i.e., *S* < *P* < *R* < *T*. If *a*_*t*_ = D, the player has not flipped the action. This MCC behavior is realizable by a value of *A* verifying *S* < *A* < *R*, *P*, *T*, which is the GRIM.

The GRIM is not exploited by an unconditional defector. In contrast, the Pavlov is exploited by an unconditional defector every other round because Pavlov players flip between cooperation and defection. In experiments, a substantial fraction of participants unconditionally defects [9, 43, 49, 50]. The parameters of the aspiration learning may have evolved such that humans behave like noisy GRIM to protect themselves against exploitation by unconditional defectors. It should be noted that the mere GRIM strategy, corresponding to *β* = ∞ and *S* < *A* < *P* in our model, does not produce MCC patterns (Fig D in S1 Text). Therefore, an involvement of reinforcement learning seems to be crucial in explaining the behavioral results, at least within the framework of the present model.

Our numerical results indicated MCC in the PGG. Past laboratory experiments using the PGG focused on CC, not MCC, to the best of our knowledge. As pointed out in previous literature [16], examining the possibility of MCC patterns in the repeated PGG with experimental data warrants future research. Conversely, applying the BM model and examining the relevance of noisy GRIM in the existing and new experimental data may be fruitful exercises.

The results were insensitive to the population structure (Fig A in S1 Text). This is in a stark contrast with a range of results in evolutionary games on networks, which generally say that the population structure is a major determinant of evolutionary game dynamics, in particular, the frequency of cooperation [51–53]. The discrepancy suggests that, under social dilemma games in laboratory experiments, humans may behave differently from the assumptions of evolutionary dynamics. In fact, regular lattices [54] and scale-free networks [16] do not enhance cooperation in behavioral experiments, which is contrary to the prediction of the evolutionary game theory. In addition, human strategy updating can considerably deviate from those corresponding to major evolutionary rules [42]. Aspiration learning provides an attractive alternative to evolutionary rules in approximating human behavior in social dilemma situations and beyond.

## Materials and Methods

### BM model for the PGG

Unlike in the PDG, the action is continuous in the PGG such that the behavior to be reinforced or anti-reinforced is not obvious. Therefore, we modify the BM model for the PDG in the following two aspects. First, we define *p*_*t*_ as the expected contribution that the player makes in round *t*. We draw the actual contribution *a*_*t*_ from the truncated Gaussian distribution whose mean and standard deviation are equal to *p*_*t*_ and 0.2, respectively. If *a*_*t*_ falls outside the interval [0, 1], we discard it and redraw *a*_*t*_ until it falls within [0, 1]. Second, we introduce a threshold contribution value *X*, distinct from *A*, used for regarding the action to be either cooperative or defective.

We update *p*_*t*_ as follows:

$$p_{t}=\left\{\begin{array}{cc} p_{t-1}+\left(1-p_{t-1}\right)s_{t-1} & (a_{t-1}\geq X\;\text{and}\;s_{t-1}\geq 0),\\ p_{t-1}+p_{t-1}s_{t-1} & (a_{t-1}\geq X\;\text{and}\;s_{t-1}<0),\\ p_{t-1}-p_{t-1}s_{t-1} & (a_{t-1}<X\;\text{and}\;s_{t-1}\geq 0),\\ p_{t-1}-\left(1-p_{t-1}\right)s_{t-1} & (a_{t-1}<X\;\text{and}\;s_{t-1}<0). \end{array}\right.$$ (3)

For example, the first line on the right-hand side of Eq (3) states that, if the player has made a large contribution (hence regarded to be C) and it has been rewarding, the player will increase the expected contribution in the next round. The stimulus, *s*_*t*−1_, is defined by Eq (2).

In the numerical simulations, we draw the initial condition, *p*_1_, from the uniform density on [0, 1], independently for different players.

## Supporting Information

S1 Text Supporting Information for: Reinforcement Learning Explains Conditional Cooperation and Its Moody Cousin. (PDF)
