# Supplementary material for: Reinforcement Learning Explains Conditional Cooperation and Its Moody Cousin
Source: PLoS Comput Biol. 2016 Jul 20;12(7):e1005034. doi: 10.1371/journal.pcbi.1005034 (PMC4954710; doi:10.1371/journal.pcbi.1005034)
Supplement: S1 Text — (PDF) [file pcbi.1005034.s001.pdf]

S1 Text
Supporting Information for: Reinforcement Learning Explains Conditional Cooperation and Its Moody Cousin

Takahiro Ezaki, Yutaka Horita, Masanori Takezawa, and Naoki Masuda

Macy-Flache Model

Macy and Flache used a variant of the BM model in the repeated PDG [32]. Their model is defined by

$$p_t = \begin{cases} p_{t-1} + (1-p_{t-1})\ell s_{t-1} & (a_{t-1} = \mathrm{C}, s_{t-1} \geq 0), \\ p_{t-1} + p_{t-1}\ell s_{t-1} & (a_{t-1} = \mathrm{C}, s_{t-1} < 0), \\ p_{t-1} - p_{t-1}\ell s_{t-1} & (a_{t-1} = \mathrm{D}, s_{t-1} \geq 0), \\ p_{t-1} - (1-p_{t-1})\ell s_{t-1} & (a_{t-1} = \mathrm{D}, s_{t-1} < 0), \end{cases} \tag{1}$$

and

$$s_{t-1} = \frac{r_{t-1} - A}{T - A}, \tag{2}$$

where p_{t-1}, s_{t-1}, a_{t-1}, and r_{t-1} are the probability of cooperation, stimulus, action, and reward (i.e., payoff), respectively, in the $(t-1)$th round. In Eq. (1), ℓ controls the learning rate and plays a similar role as β in Eq. (2) in the main text. Note that the implementation error is not included in this model.

We simulated dynamics of the BM players obeying the Macy-Flache rule in the repeated PDG on the square lattice. For three values of ℓ, the dependence of the probability of cooperation on f_{C} is shown in Figs C(a)–C(c). Similarly to the results in the main text (Fig 2), we observe CC and MCC patterns for $\ell = 0.2$ (Fig C(b)) and $\ell = 1$ (Fig C(c)). Due to the absence of the implementation error, the probability of cooperation is close to zero for $\ell = 1$ (Fig C(c)). The results for the linear fit to the relationship between the probability of cooperation and f_{C} are summarized in Figs C(d)–C(g) for various values of ℓ and A. The figures indicate that CC and MCC occur when $A < 1$ and ℓ is not small. These results are consistent with those for the BM model analyzed in the main text, including the range of A.

Noisy GRIM strategy

The noisy GRIM strategy in the two-player PDG is defined as follows [6]. If both players cooperate, the focal player will cooperate with probability $\tilde{p}_t = 1 - \epsilon$ in the next round, where $0 < \epsilon < 1/2$ is the probability of action misimplementation. Otherwise, the focal player will cooperate with probability ϵ in the next round. This action rule can be rephrased in terms of the payoff to the focal player, r_t. If $r_t = T$, R, or P, the player is satisfied and sticks to the current action (i.e., C or D) with probability $1 - \epsilon$. If $r_t = S$, the player is dissatisfied and switches the action with probability $1 - \epsilon$. The noisy GRIM action rule generalizes to the multiplayer PDG. For a given aspiration threshold A, where $S < A < P$, a player does not flip the action with probability $1 - \epsilon$ if $r_t > A$ and flips the action with probability $1 - \epsilon$ if $r_t < A$. This action rule corresponds to $\beta = \infty$ in our BM model.

The probability of cooperation conditioned on a_{t-1} is shown in Fig D for two values of A. When $a_{t-1} = \text{D}$, cooperation always occurs with probability ϵ. When $a_{t-1} = \text{C}$, cooperation occurs with a larger probability, $1 - \epsilon$, when the number of cooperators in the neighborhood, f_{C}, is at least one or two, depending on whether $(R+3S)/4 < A < P$ (Fig D(a)) or $S < A < (R + 3S)/4$ (Fig D(b)), respectively. Otherwise, cooperation occurs with probability ϵ. The binary nature of the conditional probability of cooperation does not agree with MCC patterns observed in the behavioral experiments.

Directional learning model for the PGG

In directional learning in the PGG, the direction in the previous change in the amount of contribution is reinforced if a player is satisfied. We update the expected contribution of each player as follows:

$$p_t = \begin{cases} p_{t-1} + (1 - p_{t-1})s_{t-1} & (a_{t-1} \geq a_{t-2} \text{ and } s_{t-1} \geq 0), \\ p_{t-1} + p_{t-1}s_{t-1} & (a_{t-1} \geq a_{t-2} \text{ and } s_{t-1} < 0), \\ p_{t-1} - p_{t-1}s_{t-1} & (a_{t-1} < a_{t-2} \text{ and } s_{t-1} \geq 0), \\ p_{t-1} - (1 - p_{t-1})s_{t-1} & (a_{t-1} < a_{t-2} \text{ and } s_{t-1} < 0). \end{cases} \tag{3}$$

Except for this change, the directional learning model is the same as the BM model for the PGG.

We simulated the repeated PGG in a group of four players adopting the directional learning rule. The average contribution is plotted against that of the other group members in the previous round in Figs F(a)–F(c) for three values of A. The figures do not indicate CC or MCC patterns. We did not observe CC or MCC patterns, either, when we searched a wider region in the β-A parameter space (Figs F(d)–F(g)).

Analysis of the Cimini-Sánchez model

In the Cimini-Sánchez model [25], the linear relationship between the probability of cooperation, p_t, and the fraction of neighbors that has cooperated in the previous round,

f_C, adaptively changes. We parameterize the linear relationship as $p_t = \alpha_{1,t} f_C + \alpha_{2,t}$. Variables $\alpha_{1,t}$ and $\alpha_{2,t}$ correspond to p_i^t and r_i^t (for the ith player) in Ref. [25].

Depending on the sign of the stimulus s_{t-1} and the action of the focal player in the previous two rounds, $\alpha_{1,t}$ and $\alpha_{2,t}$ are updated according to either

$$\alpha_{1,t} = \alpha_{1,t-1} + \tilde{s}_{t-1}(1 - \alpha_{1,t-1}), \tag{4}$$

$$\alpha_{2,t} = \alpha_{2,t-1} + \tilde{s}_{t-1}(1 - \alpha_{2,t-1}), \tag{5}$$

or

$$\alpha_{1,t} = \alpha_{1,t-1} - \tilde{s}_{t-1}\alpha_{1,t-1}, \tag{6}$$

$$\alpha_{2,t} = \alpha_{2,t-1} - \tilde{s}_{t-1}\alpha_{2,t-1}, \tag{7}$$

where $0 \le \tilde{s}_{t-1} \le 1$. Equations (4) and (5) imply

$$\alpha_{1,t} - \alpha_{2,t} = (1 - \tilde{s}_{t-1})(\alpha_{1,t-1} - \alpha_{2,t-1}), \tag{8}$$

which is also implied by Eqs. (6) and (7). Therefore, we obtain $\lim_{t\to\infty}(\alpha_{1,t} - \alpha_{2,t}) = 0$ except for the pathological case in which the stimulus is vanishingly small such that $\prod_{t=1}^{\infty}(1 - \tilde{s}_t) > 0$.

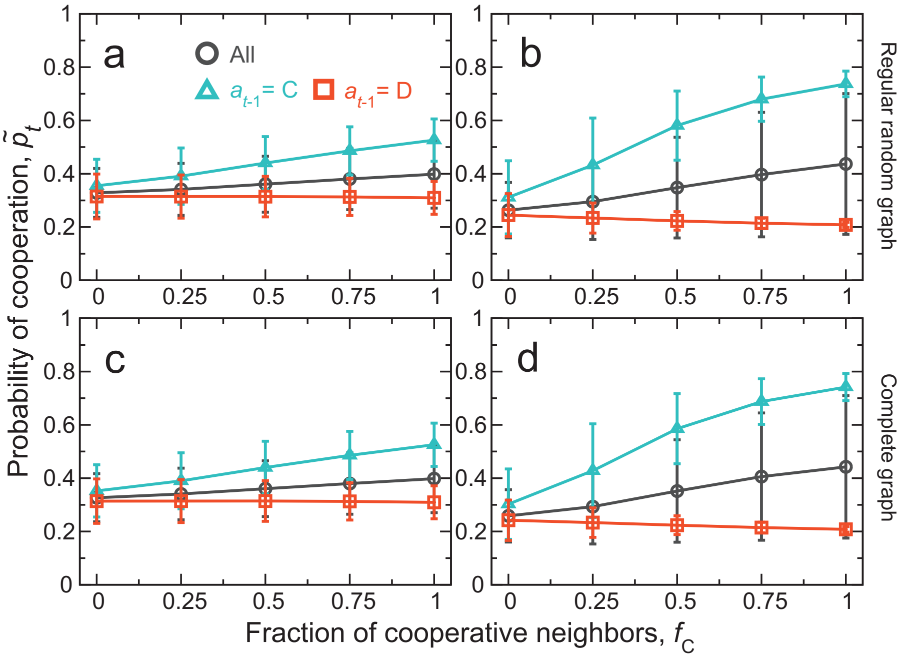

Fig A. **Repeated PDG on different networks.** (a, b) Regular random graph with $N = 100$ players and degree four. (c, d) Well-mixed group (i.e., complete graph) of five players. We set $\beta = 0.1$ in (a) and (c), and $\beta = 0.4$ in (b) and (d). We set $A = 0.5$ in all panels. See the caption of Fig 3 for the legends. The mean and standard deviation indicated by the error bars are based on 10^3 simulations in (a) and (b) and 2×10^4 simulations in (c) and (d), both yielding 2.5×10^6 samples in total.

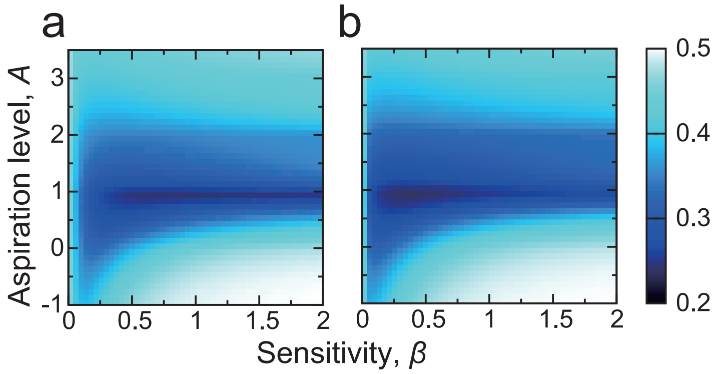

Fig B. Probability of cooperation in the repeated PDG on the square lattice averaged over the 10^2 players, first $t_{\max}$ rounds, and 10^3 simulations. (a) $\epsilon = 0.1$ and $t_{\max} = 25$. (b) $\epsilon = 0.2$ and $t_{\max} = 100$.

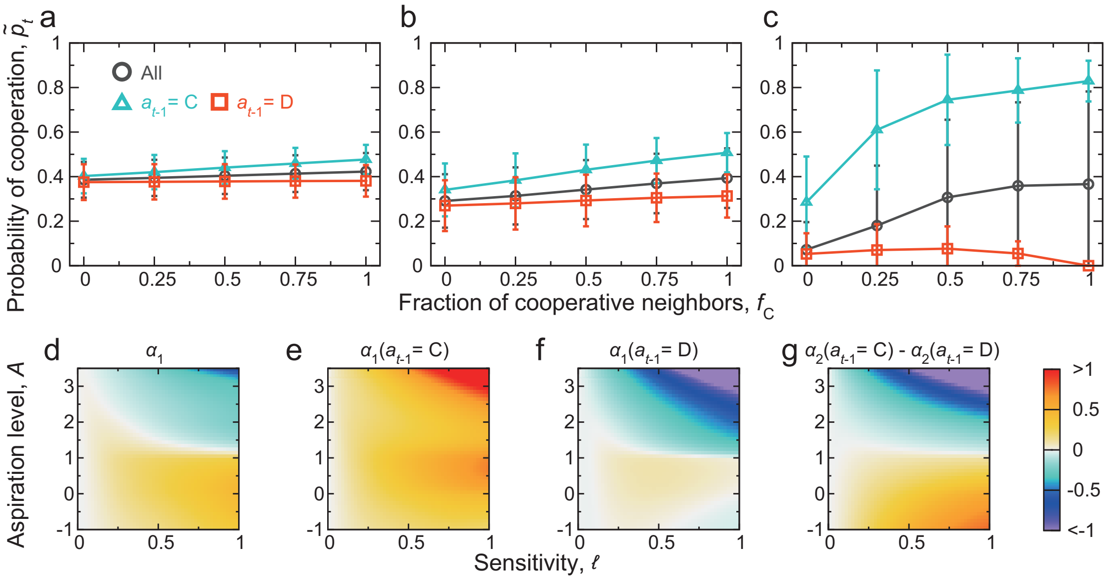

Fig C. Results for the Macy-Flache model in the repeated PDG on the square lattice. We set $A = 0.5$ in (a)–(c). Unconditional and conditional probability of cooperation is plotted against f_C with (a) $\ell = 0.1$, (b) $\ell = 0.2$, and (c) $\ell = 1.0$. See the caption of Fig 3 for the legends. (d)–(g) Slope and intercept of the linear fitting to the relationship between $\tilde{p}_t$ and f_C. See the caption of Fig 4 for the legends. For each combination of the β and A values, the linear fit was calculated on the basis of the 10^2 players, $t_{max} = 25$ rounds, and 10^3 simulations, yielding 2.5×10^6 samples in total.

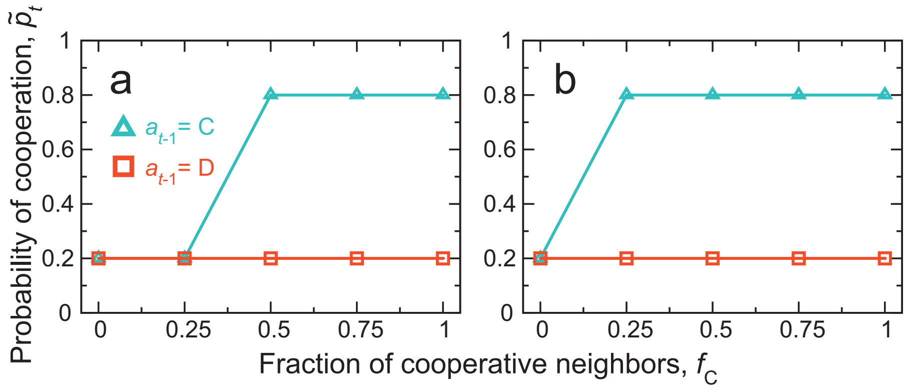

Fig D. **Probability of cooperation in the repeated PDG with the noisy GRIM strategy.** (a) $S < A < (R+3S)/4$. (b) $(R+3S)/4 < A < P$. The triangles and squares represent the probability of cooperation, $\tilde{p}_t$, conditioned on $a_{t-1} = \mathrm{C}$ and $a_{t-1} = \mathrm{D}$, respectively. We set $\epsilon = 0.2$.

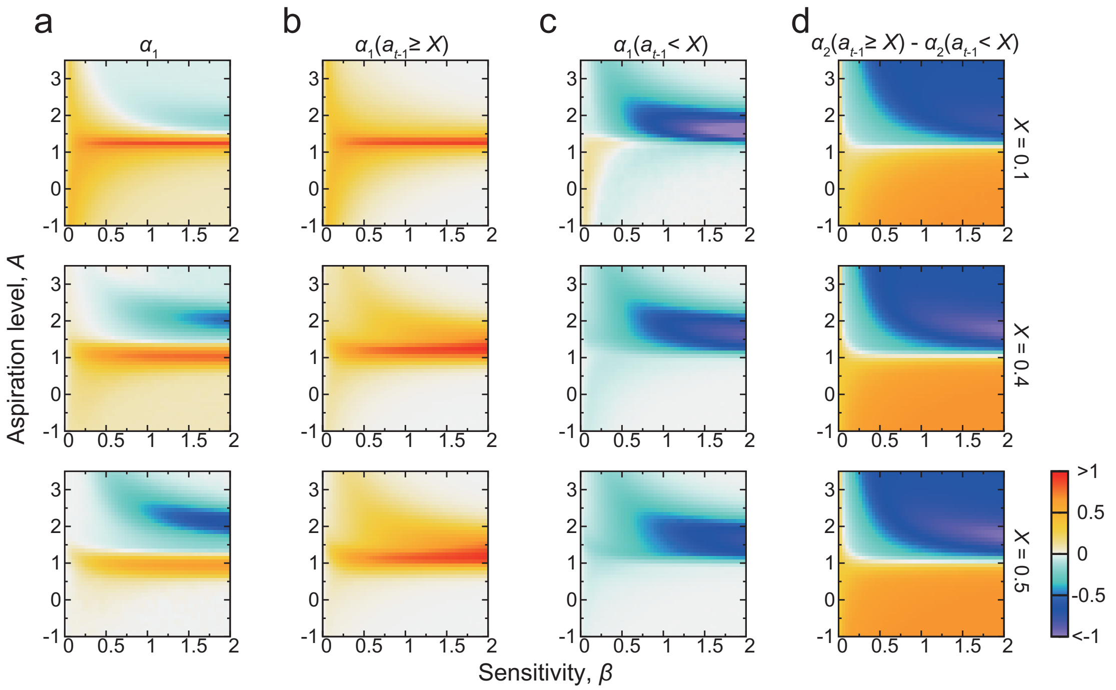

Fig E. Robustness of CC and MCC patterns with respect to X in the repeated PGG in a group of four players. (a)–(d) Slope and intercept of the linear fitting to the relationship between a_t and f_C. See the caption of Fig 5 for the legends. The results for $X = 0.1$, 0.4, and 0.5 are shown. Those for $X = 0.4$ are identical to Fig 5D–G and replicated here as a reference.

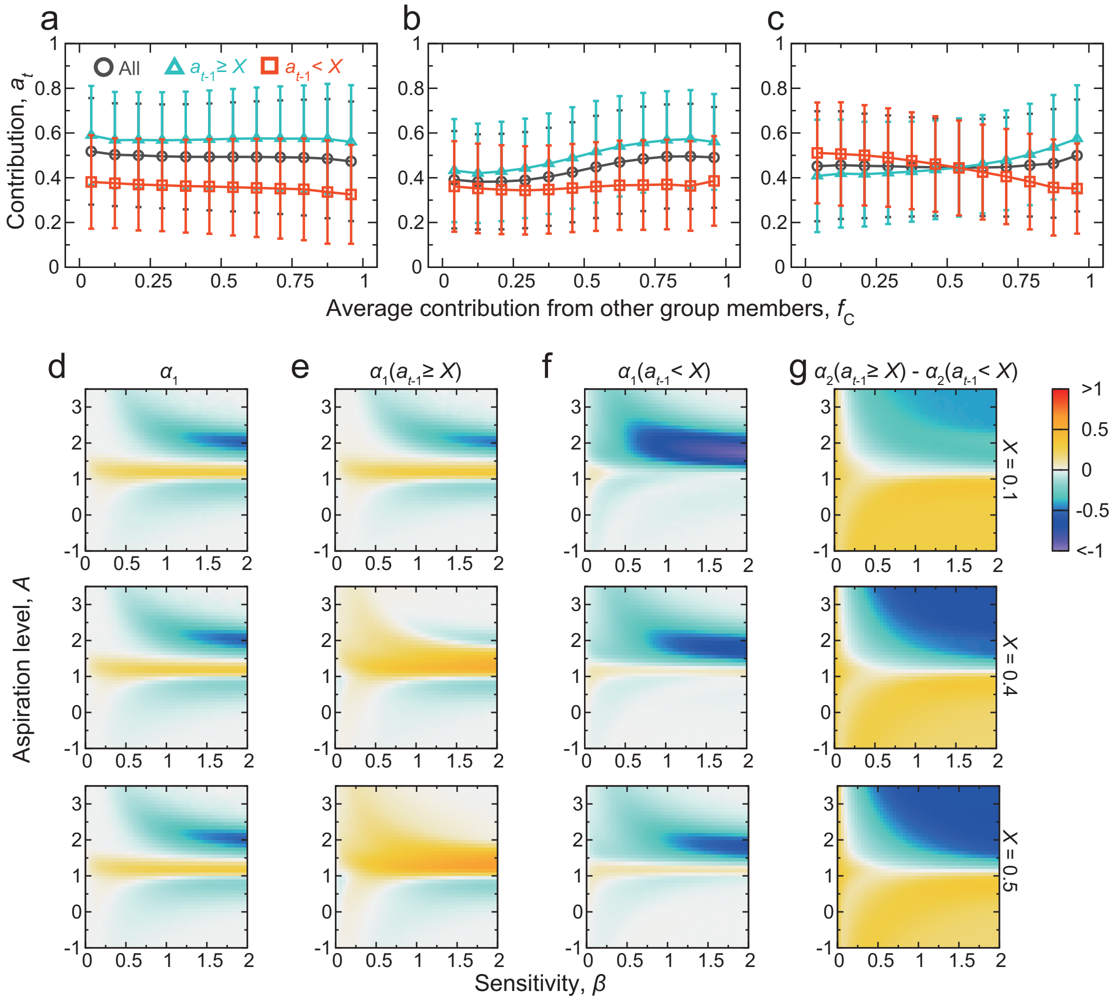

Fig F. Repeated PGG in a group of four players adopting the directional learning. (a)–(c) Contribution of a player (i.e., a_t) conditioned on the average contribution by the other group members in the previous round (i.e., f_C). We set $\beta = 0.4$. (a) $A = 0.5$, (b) $A = 1.25$, and (c) $A = 2.0$. See the caption of Fig 5 for the legends. (d)–(g) Slope and intercept of the linear fitting to the relationship between a_t and f_C. See the caption of Fig 5 for the legends. The results for $X = 0.1$, 0.4, and 0.5 are shown in (d)–(g). The mean and standard deviation in (a)–(g) and the linear fit used in (d)–(g) were calculated on the basis of the four players, $t_{\max} = 25$ rounds, and 2.5×10^4 simulations, yielding 2.5×10^6 samples in total.

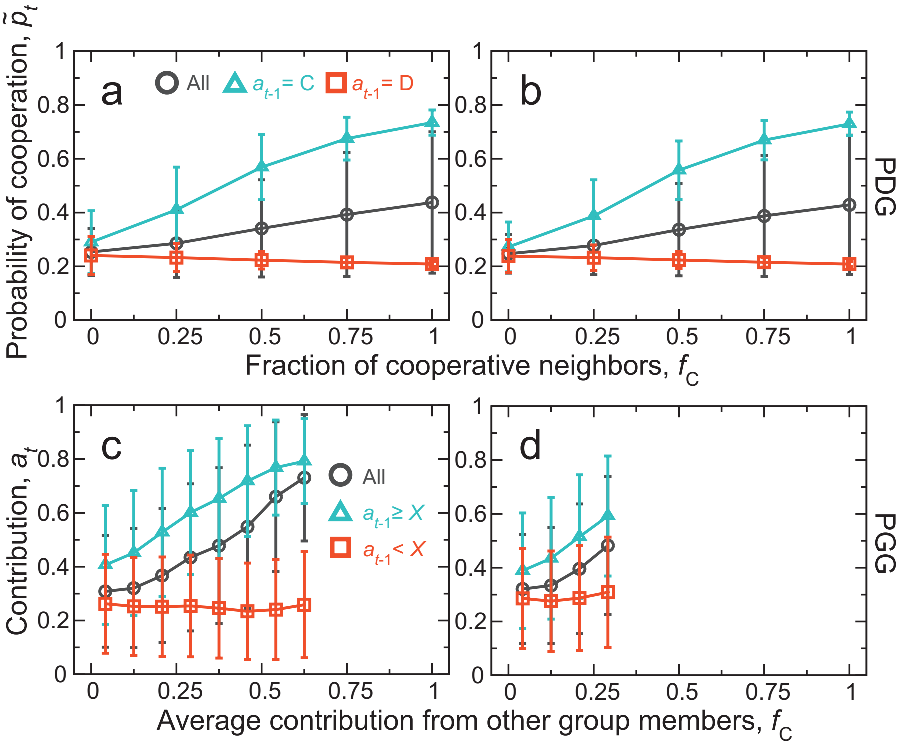

Fig G. Effects of free-riders. (a, b) Repeated PDG on the square lattice. We set $\beta = 0.4$ and $A = 0.5$. The fraction of free riders randomly assigned to the nodes is equal to (a) 0.2 and (b) 0.5. See the caption of Fig 3 for the legends. (c, d) Repeated PGG in a group of four players. We set $\beta = 0.4$, $A = 0.9$, and $X = 0.4$. The group has (c) one and (d) two free riders. Therefore, the maximum value of f_C is equal to 2/3 and 1/3 in (c) and (d), respectively. We calculated the probability of cooperation and mean contribution by excluding the free riders.
